# Virtual Screening-Accelerated Discovery of a Phosphodiesterase 9 Inhibitor with Neuroprotective Effects in the Kainate Toxicity In Vitro Model

**DOI:** 10.1021/acschemneuro.3c00431

**Published:** 2023-09-19

**Authors:** Elisa Landucci, Giovanni Ribaudo, Margrate Anyanwu, Erika Oselladore, Matteo Giannangeli, Costanza Mazzantini, Daniele Lana, Maria Grazia Giovannini, Maurizio Memo, Domenico E. Pellegrini-Giampietro, Alessandra Gianoncelli

**Affiliations:** † Department of Health Sciences, Section of Clinical Pharmacology and Oncology, University of Firenze, Firenze 50139, Italy; ‡ Department of Molecular and Translational Medicine, University of Brescia, Brescia 25123, Italy

**Keywords:** PDE9, molecular modeling, isoflavones, neurodegeneration, organotypic hippocampal slices, kainate

## Abstract

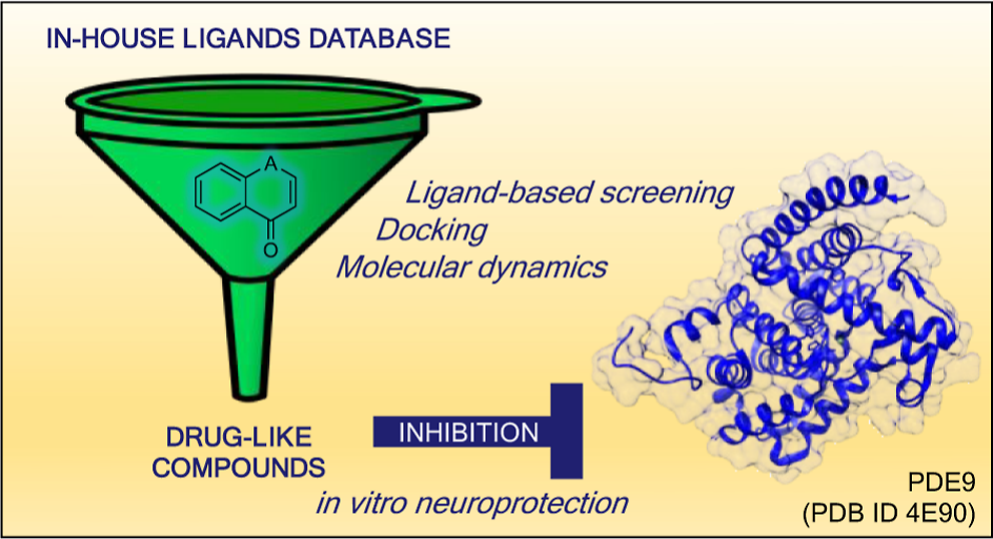

In the central nervous system, some specific phosphodiesterase (PDE) isoforms modulate pathways involved in neuronal plasticity. Accumulating evidence suggests that PDE9 may be a promising therapeutic target for neurodegenerative diseases. In the current study, computational techniques were used to identify a nature-inspired PDE9 inhibitor bearing the scaffold of an isoflavone, starting from a database of synthetic small molecules using a ligand-based approach. Furthermore, docking studies supported by molecular dynamics investigations allowed us to evaluate the features of the ligand–target complex. In vitro assays confirmed the computational results, showing that the selected compound inhibits the enzyme in the nanomolar range. Additionally, we evaluated the expression of gene and protein levels of PDE9 in organotypic hippocampal slices, observing an increase following exposure to kainate (KA). Importantly, the PDE9 inhibitor reduced CA3 damage induced by KA in a dose-dependent manner in organotypic hippocampal slices. Taken together, these observations strongly support the potential of the identified nature-inspired PDE9 inhibitor and suggest that such a molecule could represent a promising lead compound to develop novel therapeutic tools against neurological diseases..

## Introduction

Cyclic adenosine monophosphate (cAMP) and cyclic guanosine monophosphate (cGMP) represent second messengers that regulate several signaling pathways in a variety of areas of the human organism, including the processes that are related to neurobehavioral functions, memory, and cognition.^1^

Phosphodiesterases (PDEs) are responsible for the hydrolysis of cAMP and cGMP to their corresponding linear analogues, thus regulating the levels of cyclic nucleotide-based second messengers.^2^ In mammals, PDEs are classified into 11 subfamilies and are encoded by 21 different genes. Based on their specificity for cyclic nucleotides, PDEs are divided into specific to cAMP (PDE4, PDE7, and PDE8), specific to cGMP (PDE5, PDE6, and PDE9), and hydrolyzing both cAMP and cGMP (PDE1, PDE2, PDE3, PDE10, and PDE11).^3,4^

Regulatory domains and catalytic regions are highly conserved among species, while critical differences can be noted at the N- and C-terminal portions.^1^ PDEs are expressed in all tissues including brain;^4^ nevertheless, the isoforms differ for catalytic properties, subcellular localization, and sensitivity to inhibitors.

In the central nervous system (CNS), they are located in specific brain regions, and their inhibition through small molecules can promote an increase in cAMP or cGMP levels in caudate nucleus, cortex, hippocampus, and striatum.^5^ By activating protein kinase A, enhanced levels of cAMP promote CREB phosphorylation and stimulate neuronal plasticity.^6^ On the other hand, the NO/cGMP pathway induces CREB phosphorylation via protein kinase G with similar effects.^7^ Besides, recent reports in the literature are supporting the involvement of PDE inhibitors in the treatment of neurodevelopmental diseases, including autism spectrum disorders and intellectual disability.^1^ In this connection, PDE inhibitors are being investigated, developed, and repurposed to prevent and combat neurodegeneration and CNS-related diseases.^8–10^ In aging brain, an increase in PDE expression and activity and a decrease in cGMP concentration were observed.^11^

PDE5 is among the most studied families in the brain,^12,13^ but very recent reports are also supporting the role of PDE1 and PDE7 inhibitors in Alzheimer’s disease (AD) and neurodegeneration.^14,15^ More in general, brain bioavailable PDE inhibitors activating cGMP signaling are being investigated as potential therapeutic strategies against AD.^11,16^

PDE9 is endowed with high affinity for cGMP (*K*_m_ = 70 nM) and the highest selectivity over cAMP (*K*_m_ = 230 μM).^17,18^ Additionally, due to its specific localization and activity, PDE9 is receiving increasing attention as a target for the development of neuroprotective agents.^19–21^ A widespread localization of PDE9 mRNA was observed in a number of human brain areas,^22^ and PDE9 mRNA were expressed in different regions of hippocampus in both rats and mice.^23^ PDE9 mRNA expression/localization highly changes across neurodevelopment in mice and humans, and it is elevated in the aged human hippocampus with dementia.^24^ In preclinical studies, the selective PDE9 inhibitor BAY73-6691 increased basal synaptic transmissions and enhanced early long-term potentiation (LTP) in hippocampal slices obtained from old rats,^25^ while another investigational PDE9 inhibitor, named PF-04447943, improved indicators of hippocampal synaptic plasticity and cognitive function in a variety of cognition models in rodents.^26^ PF-04447943 improved memory, LTP, and hippocampal spine density in the Tg2576 FAD mouse model; recently, many clinical studies were performed to investigate safety, tolerability, pharmacokinetic, and pharmacodynamic properties of PDE9 inhibitors in multiple subjects including healthy participants and patients with schizophrenia, psychotic disorders, and AD.^5^ In phase 2 clinical trials, PF-04447943 did not improve cognition over placebo in mild to moderate probable AD.^27^ A novel PDE9 inhibitor, BI-409306, recently completed phase 1 clinical trials and entered phase 2 trials in patients with prodromal (study 1) or mild to moderate AD (study 2).^28^

Recently, we identified cannabidiol (CBD) as a drug-like PDE9 inhibitor by virtual screening and in vitro experiments.^29^ Importantly, CBD was approved by Food and Drug Administration (FDA) in 2018 and by European Medicines Agency (EMA) in 2019 as an add-on antiepileptic drug in 2 year-old children with Dravet syndrome and Lennox-Gastaut syndrome. In a previous study, we have also demonstrated that CBD consistently reduced neuronal damage induced by kainate (KA).^30^ It must also be considered that the role of cAMP/cGMP signaling has been well established in the context of seizures and epilepsy.^31,32^

Based on this scientific knowledge, it has been hypothesized that PDE9 may be involved in such disorders. Thus, the aim of the current study is to pave the way for the identification of drug-like PDE9 inhibitors with a potential application against this CNS disease. To reach this goal, computational tools [virtual screening, docking, and molecular dynamics (MD)] and experimental techniques were combined, and a candidate was highlighted and tested in rat organotypic hippocampal slices exposed to KA, a widely accepted in vitro epileptic model.^30,33,34^

## Results and Discussion

### Identification of PDE9 Inhibitors by Ligand-Based Virtual Screening

In our search for potential PDE9 binders, we took advantage of our in-house database comprehending more than 1600 small molecules, which were synthesized and characterized by our research group over the years. The first step for the preparation of the virtual library consisted in the evaluation of the druglikeness of the molecules and of their predicted pharmacokinetic properties. The database was therefore imported in Schrödinger Maestro and analyzed with the QikProp tool embedded in the software after the preparation of the compounds with LigPrep. Only the molecules bearing a maximum of one violation to the “Lipinski’s rule of five” was admitted to the following step of investigation, and in particular, the criteria that were adopted are reported as follows: molecular weight <500 g/mol, log *P* < 5, number of hydrogen bond donors ≤5, and number of hydrogen bond acceptors ≤10.^35^

Natural or nature-inspired compounds have been widely demonstrated to possess neuroprotective action, and it is well accepted that this behavior is associated with the inhibition of one or more PDE isoforms.^2,36–38^ In the past, attention has been dedicated to caffeine, theophylline, papaverine, and other nonspecific alkaloids, but over the years, the focus has been moved to flavonoids, although they were mostly associated with their role in inhibiting PDE5.^39–42^ In this context, our group recently proposed a set of in silico tools to evaluate the effects of flavonoids and other natural compounds in the inhibition of various PDEs involved in neurodegenerative disorders.^43^ This first study showed indeed that flavonoids represent a promising class of compounds targeting PDE9, and the results set the basis for a subsequent in silico analysis in which a small library of compounds was screened toward PDEs showing, again, improved affinity toward PDE9.^44^

**Figure 1:**
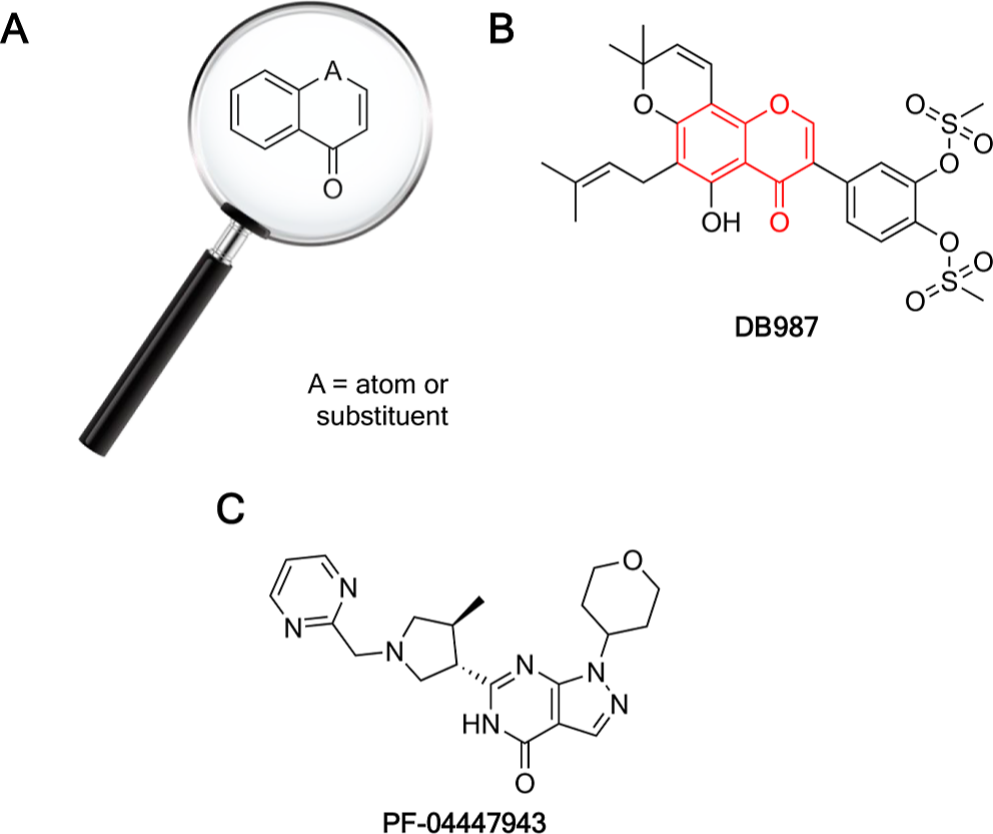
Chemical structure of the chromone scaffold used for the virtual screening procedure (A). Chemical structure of DB987, the compound highlighted by the screening, in which the red structure represents the part of the molecule matching query (B). Chemical structure of the reference compound, PDE9 inhibitor PF-04447943 (C).

Based on such results and on other data from the literature,^21^ our in-house database was filtered using a structural query, in order to highlight the compounds bearing a chromone scaffold, which is the core of flavonoids, using a ligand-based approach. Additionally, the oxygen in position 1 of the pyran ring was allowed to be substituted to also include other natural or nature-inspired compounds such as naphthoquinones and anthraquinones (Figure 1). As a result, we obtained a subset of 441 compounds that were processed using the Schrödinger suite and docked with Glide to the 3D structure of PDE9. The 20 most promising compounds in terms of docking score were admitted to a subsequent experimental solubility test, which was performed in water and in PBS buffer. Compound solubility is a crucial aspect that should be considered in drug discovery studies: we introduced this fast and cost-effective step in the screening protocol to further refine computational data, building at the same time a bridge toward in vitro experiments.

Considering the results of the virtual screening and of the solubility test, we decided to analyze more in detail the isoflavone DB987 (Figure 1).

Interestingly, the identified compound can be defined as a semisynthetic molecule. Indeed, the isoflavone DB987 can be obtained through the modification of pomiferin, a natural flavonoid extracted from *Maclura pomifera*.^45^

### Structure of PDE9 and Molecular Docking

From a structural point of view, the catalytic domain (C domain) of PDE9 is constituted by 16 helices (amino acids 181–506), and it associates in dimers where the active site of one monomer is totally exposed to the solvent, while the other is hindered by the C-terminal helix of the neighboring one.^21,46^ A monomeric catalytic domain is composed of pockets, three in total, and with different features, namely, metal binding (M), hydrophobic clamp (H), and glutamine pocket (Q).^44,47^ Each of these pockets has one or more residues that are important for the ligand and enzyme interaction and, consequently, for the inhibition of the latter. Putative key residues for PDE9 are Gln453 (Q pocket) and Phe456 since it is reported that through interactions like hydrogen bonds and π–π stacking interactions, they could lead to inhibitory effects.^46,48^ Furthermore, other relevant residues have been identified as important targets to increase the inhibitory capacity of ligands. Some of them are Phe251, His252, Asp300, Met365, Asp405, Leu420, Phe441, and Phe456.^49^

Then, as PDE9 is a metalloprotein, its catalytic action depends on two divalent cations, such as zinc (Zn^2+^) and magnesium (Mg^2+^). Zn^2+^ is reported to form coordination bonds with residues in the protein side chain like Asp402, Asp293, His292, and His256 and two water molecules, while Mg^2+^ is coordinated by Asp293 and five water molecules.^18,21,50^

The 3D structure consisting of a crystallized PDE9 enzyme and a cocrystallized PF-04447943 ligand was retrieved from the RCSB Protein Data Bank (PDB ID: 4E90).^51^ In the context of this structure, it can be noted that PF-04447943 established hydrophobic interactions with some of the residues present in the hydrophobic clamp of PDE9. The aromatic ring present in Phe456 established three π–π stacking interactions with pyrazole, the hydroxy-substituted pyrimidine ring, and the pyrimidine moiety (4.29, 3.70, and 5.08 Å, respectively). Two hydrogen bonds were identified between Gln453 and the hydroxy-substituted pyrimidine of the ligand. The first (1.75 Å) occurs between N7 and the carbonylic oxygen in Gln453 and the other (2.12 Å) between O6 present in the pyrimidine of the ligand and –NH_2_ in the amino acid.

**Figure 2:**
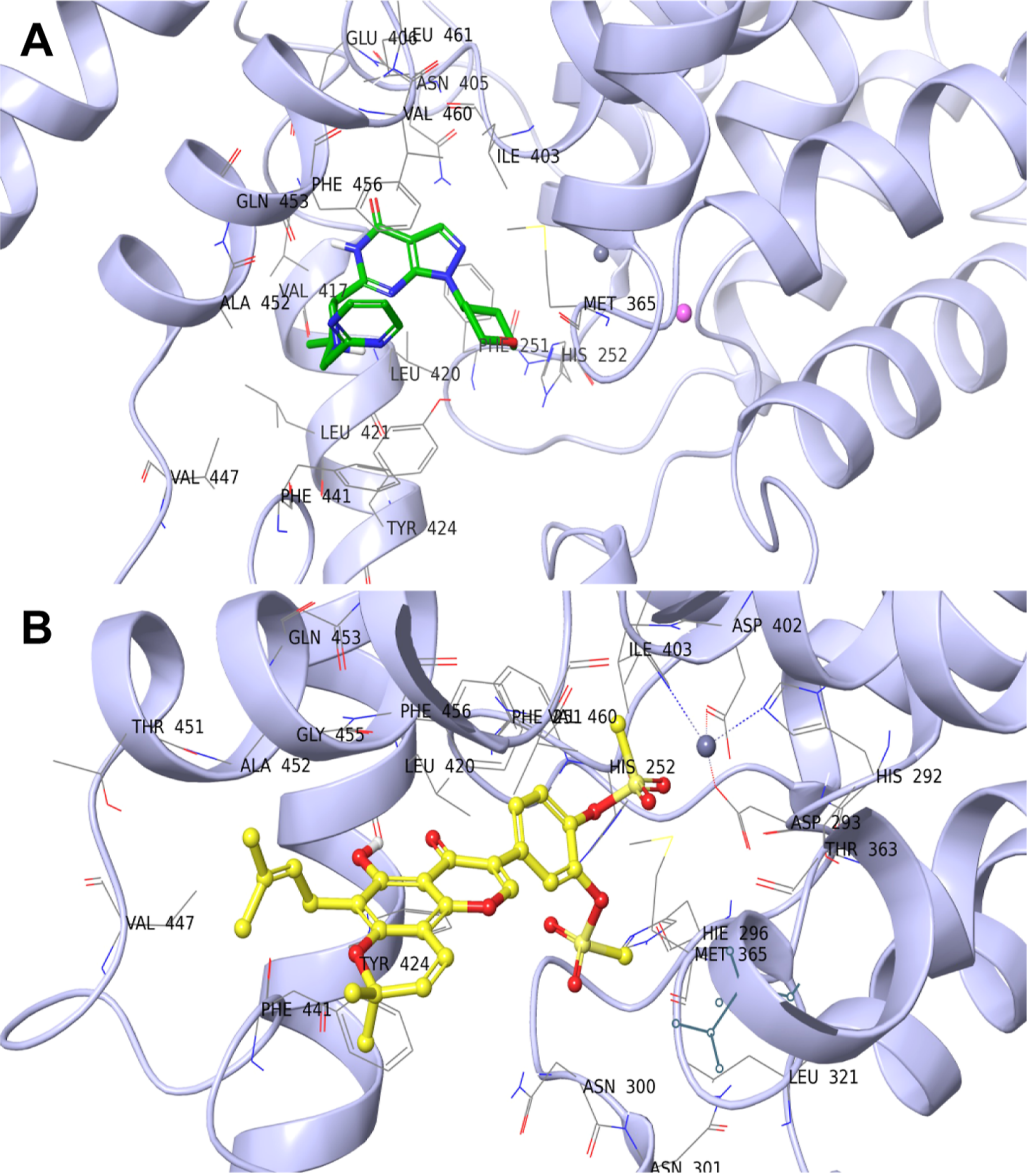
Binding poses for PF-04447943 (A) and DB987 (B) in the predicted interaction pattern with PDE9. The residues at the interaction distance (<5 Å) within the binding pocket have been labeled.

The complexes obtained from molecular docking analysis were evaluated from the perspective of the ligand–PDE9 interactions. Redocking of the ligand (−9.8 kcal/mol, Figure 2A) reproduced the original pose, and the root-mean-square deviation (rmsd) value with respect to the cognate ligand, which should be at most 2.50 Å,^52^ was of 1.18 Å, thus indicating that the method is accurate. For comparison, the docking pose of DB987 is reported in Figure 2B.

Since Gln453 and Phe456 are considered in the literature to be some of the important residues for achieving inhibitory effects of the ligand against the enzyme,^48,53,54^ their interaction with the identified compound was studied. More specifically, interaction analysis revealed that hydrogen bonds, π–π stacking interactions, salt bridges, and metal coordination systems were present in the evaluated complex.

More specifically, isoflavone DB987 (−11.7 kcal/mol, Figure 2B) established two hydrogen bonds and one metal coordination bond. These were generated, respectively, with O38 and –OH in Tyr424 (2.06 Å) and between O37 and –NH_2_ concentrations in Asn300 (2.67 Å). One metal coordination bond (2.13 Å) with Zn^2+^ and O32 was also detected.

### MD Simulations

**Figure 3:**
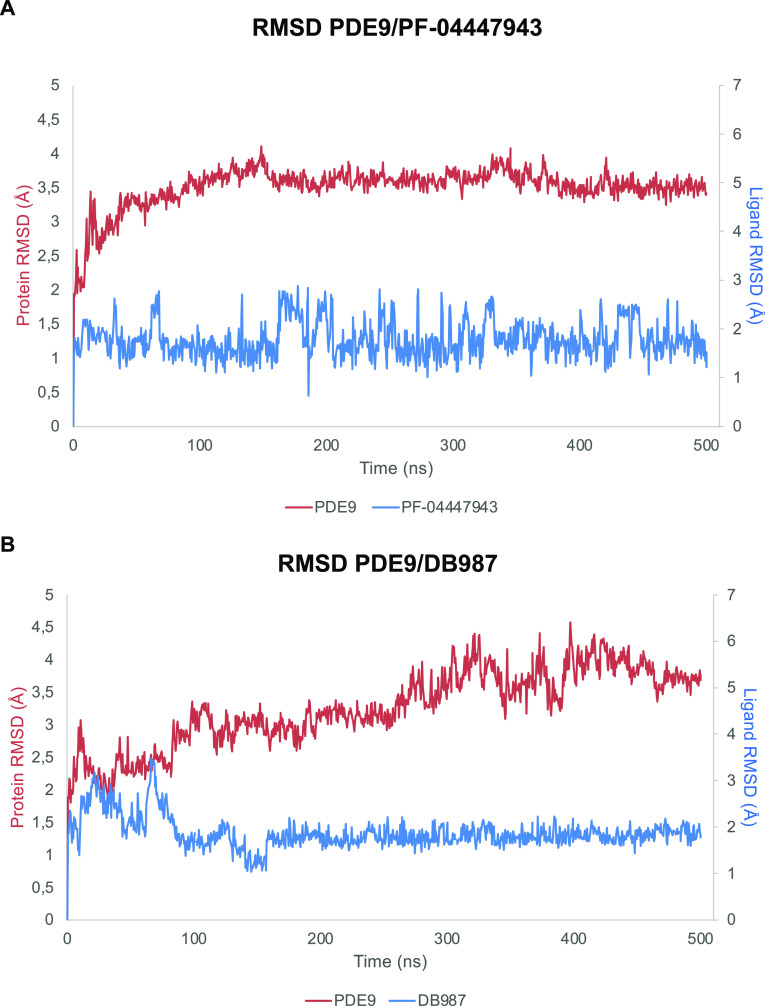
Trajectories retrieved from MD simulations showing the rmsd of the PDE9/ligand systems over the simulation time (A: PF-04447943, B: DB987). The plots for the heavy atoms of the ligand are depicted in blue (aligned with the protein), while the plots for protein C-alphas are depicted in red.

To study the stability of the interaction predicted by molecular docking in a more accurate fashion, MD studies were performed. The starting point for these simulations was, in both cases, the best-docked poses from molecular docking. The dynamics simulation showed that most of the interactions present in best-docked poses were confirmed and maintained over time, even with different contributions. The studied PDE9/ligand complexes achieved stable MD trajectories within the simulation time frame (500 ns, Figure 3).

First, the binding mode of PF-04447943 was analyzed. The stability of the receptor was achieved after 150 ns (Figure 3A). Even in the case of the ligand, the rmsd trajectory was observed to be stable, except for a few sections of the simulation in which it showed some fluctuations. This could be explained by the measurement of root-mean-square flexibility (RMSF) value (Figure S1A), which shows that the pyrimidine ring and tetrahydropyran portions of the ligand, being bound to the scaffold by a single bond, have a larger degree of freedom. This is also confirmed by Figure S2, in which we reported the rmsd plot related only to the scaffold of the ligand that, being more rigid, shows very little fluctuation and is retained in place throughout simulation. The behavior of the protein was also considered, and as expected, the most flexible components of the structure turned out to be the C- and N-terminal regions and the side chains containing most of the residues that establish bonds with the ligand.

In addition to the stability and flexibility of the system, the preponderant interactions established between the ligand and PDE9 during the simulation were also evaluated. The hydrogen bond between the carbonylic oxygen atoms of Gln453 and N7 observed in the docked model is maintained over time. Subsequently, Asn405 also established one hydrogen bond with O6 of the previous moiety. Then, Phe456 then establishes a π–π stacking interaction with the hydroxy-substituted pyrimidine ring of allopurinol and creates a π-cation interaction with –N13. Other less preponderant hydrophobic interactions such π–π stacking interaction, π-cation, and others like van der Waals have been identified among residues such as Phe251, Met365, Ile403, Leu420, Leu421, Tyr424, Phe441, and Phe456. An overview of the established interactions is reported in Figures S3–S5.

The MD profile of the PDE9/DB987 complex was then studied (Figure 3B). Unlike in the case of the PDE9/PF-04447943 complexes, system stability is achieved after about 100 ns according to rmsd plots. Looking at the stability of the ligand, some spikes were again observed in the rmsd curve. This may be due to the presence of the single bond between C6 and C7, which introduces more freedom to C7–C11 groups (Figure S1B). Concerning the flexibility of PDE9 verified by RMSF, as in the two previous cases, the C- and N-terminal parts and the areas of the side chains interacting with the ligand are the most flexible.

With the aim of assessing that in addition to being stable, the ligand was also capable of establishing interactions with the receptor; the latter have been evaluated using the same threshold value adopted for PF-04447943 (see Methodssection). One hydrogen bond between Met365 and O38 is present. The other hydrogen bonds detected below the threshold value are those established with residues His296, Asn300, Asn301, and Met365.

In the case of DB987, hydrophobic interactions such as π–π stacking, π–cation interaction, and others such as van der Waals were observed during the simulation. For instance, Phe456 established one π–π stacking interaction with one of the aromatic rings of the ligand. Less predominant hydrophobic interactions such as those with Leu321, Met365, Ile403, Val417, Leu420, Leu421, Tyr424, Phe441, Ala452, and Phe459 were present. The analysis also reported the presence of ionic interactions due to the presence of metal coordination centers. Zn^2+^ formed metal coordination bonds with His296, His292, Asp402, Asp403, and O32 of DB987 during the simulation. Mg^2+^, on the other hand, established bonds of the same nature with residues His292, Asp293, Glu322, His325, and Thr363 and with the O31 of the ligand. An overview of the established interactions is reported in Figures S3–S5.

### Assessment of PDE9 Expression in Organotypic Hippocampal Slices

**Figure 4:**
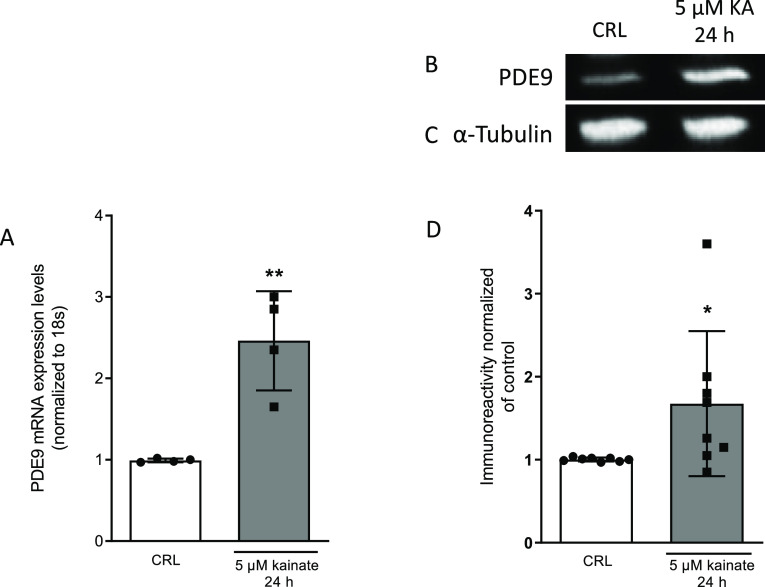
PDE9 increased in organotypic hippocampal slice cultures exposed to KA. The mRNA levels of PDE9 were increased in slices treated with KA (A). Illustrative blots using antibodies directed against PDE9 (B) and β-tubulin (C). Quantitative analysis of immunoreactive bands shows significant changes in the levels of PDE9 in slices treated with KA (D). Dot blots show the results of four (A) and eight (D) experiments from independent slice preparations, and each dot is the pool of four slices. *p < 0.05 and **p < 0.01 vs CRL (unpaired *t*-test).

The expression of PDE9 was detected in organotypic hippocampal slices with or without exposure to KA. The gene and protein expression of PDE9 was elevated by PCR real time and western blot, as shown in Figure 4, where we observed a significant increase in the level of PDE9 in slices exposed to 5 μM KA for 24 h (Figure 4C,D). Previous studies showed that PDE9 mRNA is elevated in the aged human hippocampus with dementia when there is a history of traumatic brain injury,^24^ and PDE9 expression is upregulated during cardiac hypertrophy and heart failure.^55^

**Figure 5:**
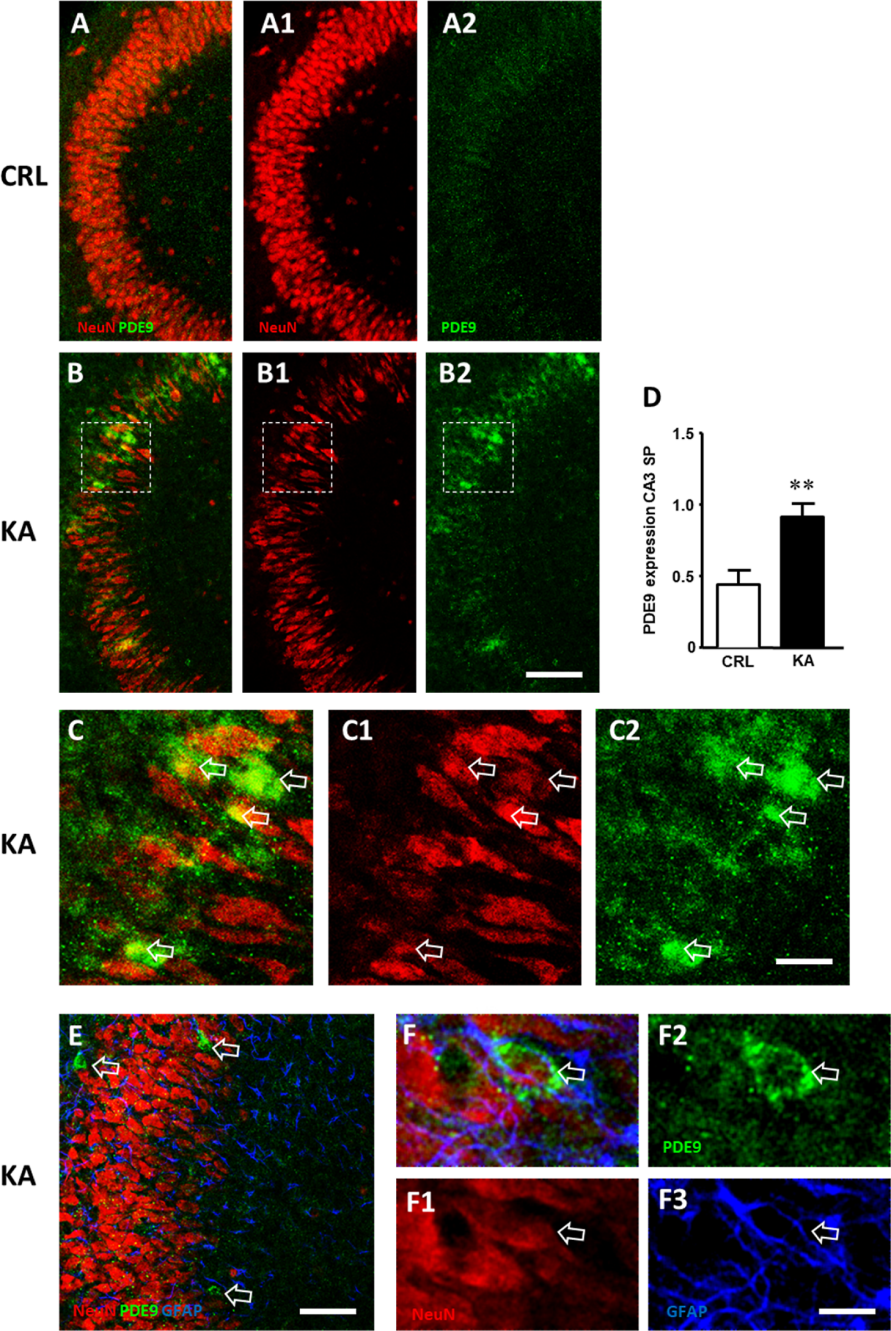
(A-B2) Immunohistochemical assessment of neuronal damage and PDE9 expression in the CA3 hippocampus of organotypic slices after treatment with KA. (A1,B1) Representative confocal images of fluorescent immunostaining of NeuN-positive neurons in area CA3 of CRL (A1) and KA (B1) slices. (A2,B2) Representative confocal images of fluorescent immunostaining of PDE9 in area CA3 of CRL (A2) and KA-treated slices (B2). (A, B) Merge of the previous images. All images were captured with a 20× objective. Scale bar: 100 μm. (C-C2) Magnifications of the framed areas of the corresponding slice shown in B, B1, and B2. (C1) Immunostaining of NeuN showing the presence of damaged neurons with an elongated, shrunk cytoplasm. (C2) Immunostaining of PDE9 showing the expression of the enzyme in many CA3 pyramidal neurons (open arrows). (C) Merge of the two previous images (open arrows indicate neurons positive for PDE9 immunostaining). Scale bar: 25 μm. (D) Quantitative analysis of PDE9 immunostaining in CA3 SP (CRL *n* = 10 and KA *n* = 9). Statistical analysis: Student’s *t*-test: **P < 0.01 KA vs CRL. (E-F3) Representative confocal images of triple immunofluorescent labeling of neurons (NeuN, red), PDE9 (green), and astrocytes (GFAP, blue) in CA3 of KA-treated slices captured with a 40× objective. Open arrows indicate PDE9-positive pyramidal neurons. No colocalization with astrocytes was found. Scale bars: 70 μm (E) and 15 μm (F-F3).

Using a different experimental technique, we assessed the effect of exposure to 5 μM KA for 24 h on the expression of PDE9 in the CA3 hippocampus by immunofluorescent staining and confocal microscopy. The qualitative confocal microscopy images (Figure 5A-B2) show that PDE9 expression was increased in CA3 SP of slices treated with KA (Figure 5B-B2) in comparison to CRL slices (Figure 5A-A2). As already published,^30^ in KA-treated slices, the morphology of neurons appeared altered, and numerous neurons showed a shrunk, elongated cytoplasm in comparison to controls, and we also observed morphological alterations on glia cells (see Figure S6A–E in the Supporting Information). The confocal images shown in Figure 5D-D2, magnifications of the framed areas shown in Figure 5B-B2, clearly demonstrate that PDE9 expression increased in CA3 pyramidal neurons that show signs of damage (Figure 5C-C2, open arrows).

The quantitative analysis (Figure 5D) demonstrated that the treatment with KA significantly increased PDE9 expression in CA3 SP (Student’s *t*-test: **P < 0.01 KA vs CTR; CTR, *n* = 10; and KA, *n* = 9).

In addition, triple immunostaining demonstrated no colocalization of PDE9 expression with astrocytes in CA3 SP or SR in KA-treated slices (Figure 5E-F3). Furthermore, no colocalization of PDE9 with microglia was present in CA3 in organotypic slices treated with KA (Figure S6F in the Supporting Information). Consistent results from different approaches indicated that PDE9 may play a key role in brain degeneration.

These data are in accordance with previous studies where PDE9 was localized only in neuron cells.^23^

### Assessment of PDE9 Inhibitory Activity

**Figure 6:**
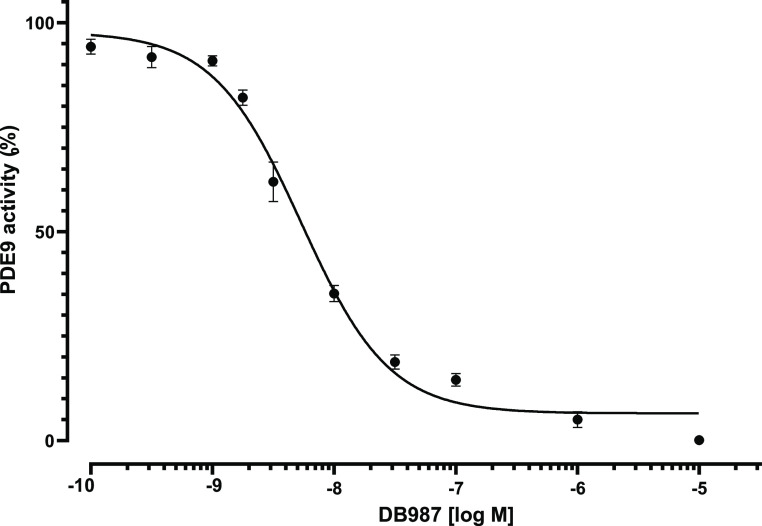
Concentration-dependent inhibitory activity of DB987 on PDE9 in vitro. Results are shown as the mean of eight replicates ±SEM.

We already reported the inhibitory activity of CBD on PDE9 in vitro (IC_50_ = 110 nM).^29^ In the current study, the potential of DB987 was tested under the same conditions. Experimentally, the PDE9 activity was evaluated in the presence of increasing concentrations of the tested compound in an enzymatic assay, and the results were converted into percentages to calculate the IC_50_ values (Figure 6).

An IC_50_ value of 5.35 nM was calculated for DB987. The inhibitor potency resulted very similar to that earlier observed with PF-04447943, which was adopted as a positive control in agreement with our previous studies, and the newly identified compound outperformed CBD in terms of IC_50_.^29^

### Effects of PDE9 Inhibitors on KA Neurotoxicity in Organotypic Hippocampal Slices

**Figure 7:**
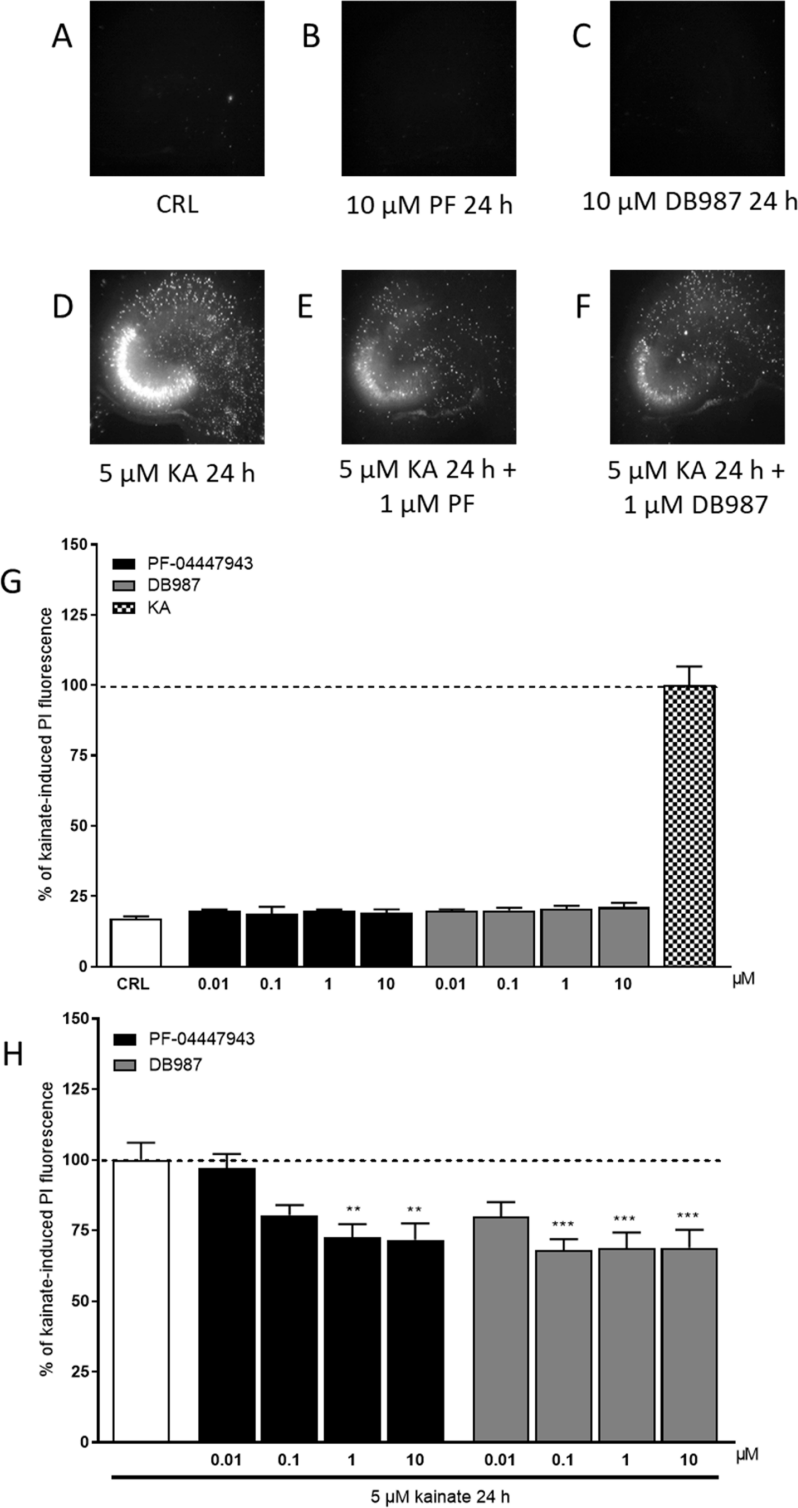
Qualitative and quantitative analyses of the effects of PDE9 inhibitors in rat organotypic hippocampal slices under normal conditions or exposed to KA. (A) Hippocampal slice under normal conditions (background PI fluorescence), (B) slice exposed to 10 μM PF for 24 h, (C) slice exposed to 10 μM DB987 for 24 h, (D) slice exposed to 5 μM KA for 24 h displaying intense PI labeling in the CA3 subregion, and (E,F) CA3 damage induced by KA was attenuated by the presence of 1 μM PF and DB987. (G) PDE9 inhibitors alone did not induce side effects. (H) PDE9 inhibitors significantly attenuated CA3 damage in a dose-dependent manner. Bars represent the mean ± SEM of at least five experiments run in quadruplicate. **p < 0.01 and ***p < 0.001 vs KA (one-way ANOVA plus Dunnett’s test).

In our previous studies, we had observed that CBD reduced the damage induced by KA in the CA3 regions of organotypic hippocampal slices, as evaluated by PI fluorescence,^30^ and we identified CBD as a drug-like PDE9 inhibitor by virtual screening and in vitro experiments.^29^ In this study, we observed that the studied PDE9 inhibitors did not induce damage in the CA3 area of slices at different concentrations (Figure 7A,B,C,G).

When the slices were incubated for 24 h with 5 μM KA, we observed a selective damage in the CA3 area (Figure 7D), in agreement with previous findings.^30,34^ Treatment of slices with the PDE9 inhibitor PF-0447943 (0.01–1 μM) during the 24 h KA exposure significantly reduced neuronal death in a dose-dependent manner in CA3 at concentrations of 1 and 10 μM (Figure 7E,H). Importantly, we observed similar neuroprotective effects when the slices were incubated with the new PDE9 inhibitor DB987 (0.01–10 μM) that reduced the KA-induced damage at the concentrations of 0.1–10 μM (Figure 7F,H) in accordance with the IC_50_ values obtained by the enzymatic assay.

## Conclusions

Computational studies were performed to evaluate the predicted binding affinity, stability, and interactions of a novel potential PDE9 ligand by molecular docking and MD simulations.

In particular, molecular docking analyses showed that the highlighted semisynthetic compound, namely, the isoflavone DB987, interacts efficiently with the PDE9 catalytic site and MD simulations confirmed the stability of the complex formed by the ligands.

Then, we experimentally demonstrated that PDE9 was expressed in rat organotypic hippocampal slices and increased upon exposure to KA. Most importantly, the compound showed nanomolar inhibitory activity on PDE9 and was able to reduce the neuronal damage induced by KA in vitro.

Taken together, the results of this study pave the way for more targeted development of new, semisynthetically obtainable, potential PDE inhibitors to be used against neurodegenerative diseases.

## Methods

### Database Search

The considered database is constituted of 1632 compounds synthesized in-house. The database file, in SDF format, was directly loaded in Schrödinger Maestro version 13.4.134, MMshare Version 6.0.134 Release 2022-4, Platform Linux-X86_64: Maestro (Schrödinger, LLC, New York, NY, USA 2021). The structures were prepared with the LigPrep tool under the OPLS4 force field using an Epik ionizer;^56–58^ all the ionization states were generated in the 7 ± 2 pH range including the possible metal states leading to a total of 3150 molecules, which were analyzed with the QikProp tool to predict the pharmacokinetic properties. The molecules having more than one violation to the “Lipinski’s rule of five”^35^ were discarded, reducing the number of possible hits to 1762. These molecules were then filtered based on their chemical structure, and to perform this step, we loaded the SDF file retrieved from Schrödinger in ChemFinder Version 20.1.1 (ChemOffice, PerkinElmer Informatics, Inc. Waltham, MA, USA, 2020), and we used the chromone-based query (Figure 1) to screen the compounds, which were reduced to 441. Then, the molecules were loaded into Schrödinger to perform the docking study as described in the following paragraphs. The complexes resulting from docking experiments were visually inspected, and the 20 molecules with the best poses, also considering the docking score, were selected. The hit compounds that were physically retrieved from the database following the preliminary virtual screening were subjected to a solubility test. A 10 mM stock solution of the compounds was prepared in DMSO, and they were then diluted to 100 μM solutions in H_2_O. Then, three more 1:10 dilution steps were performed in phosphate-buffered saline (PBS, pH 7.4) up to 1 nM to assess adequate solubility of the compounds in the concentration range considered in in vitro test.

### Molecular Docking

Human PDE9 3D structure was obtained from the RCSB Protein Data Bank (PDB, http://www.rcsb.org, access date 30 January 2023). PDE9 in complex with inhibitor PF-04447943, entry 4E90, was selected (resolution 2.50 Å).

The preparation step was performed through the Protein Preparation Workflow tool. Default parameters were selected, for example, adding hydrogens, assigning disulfide bonds, adding missing side chains using Prime, removing waters, adjusting charges, and minimizing the convergence of heavy atoms to rmsd under the OPLS4 force field.^57^

Ligands were prepared for docking with the LigPrep tool under the OPLS4 force field. Possible ionization and metal binding states at target pH 7.0 ± 2.0 were generated with an Epik ionizer.^58^

The binding poses of PF-04447943 and DB987 in complex with PDE9 were obtained by molecular docking calculations using Glide.^59,60^ Ligands and receptors were prepared as described in the previous paragraphs.

The docking protocol consisted of rigid receptor and flexible ligand sampling, which was performed under the OPLS4 force field^56^ using standard precision (SP) with default settings like 0.80 scaling factor for the ligand atom van der Waals radius, 0.15 partial range cutoff, adding Epik state penalties to the docking score, generation of one pose per ligand, and performing postdocking minimization for generated poses.

The receptor grid was prepared with the Receptor Grid Generation tool included in Schrödinger, with default settings.^60^ After the identification of the best poses, the root-mean-square deviation (rmsd) value was used to validate the adopted method with the rmsd.py script, and a score under 2.50 Å was admitted to consider the analysis sufficiently accurate. In the case of this study, a rmsd value of 1.18 Å was obtained. The obtained poses were evaluated according to the GlideScore (GScore) scoring function, which is expressed in −kcal/mol, and through the inspection of the interactions established between ligands and PDE9. For interaction analysis, only residues within 5 Å in the binding pocket were considered.

### Molecular Dynamics

MD simulations were performed using the GPU-accelerated Desmond tool of Schrödinger LLC (Schrödinger Release 2023-1: Desmond Molecular Dynamics System, D. E. Shaw Research, New York, NY, USA, 2021. Maestro-Desmond Interoperability Tools, Schrödinger, New York, NY, USA 2021).^61^ The best protein–ligand complexes retrieved from the SP molecular docking step were used to prepare the solvated system to perform MD.

By using System Builder,^61^ all the three complexes were solvated using the explicit TIP3P water model. Periodic boundary conditions were set with an orthorhombic box shape of 10 × 10 × 10 Å. During ion placement, Na^+^ ions were used to neutralize the system, a concentration of 0.15 M of NaCl was set, and an exclusion zone with 20 Å from the ligand was added. A run of 500 ns was performed for each complex under the OPLS4 force field with the temperature set at 300 K and pressure at 1.0 bar. Further, the Martyna–Tuckerman–Klein chain coupling scheme with an isotropic coupling constant of 2.0 ps for the pressure control and the Nosé–Hoover chain coupling scheme for the temperature control (NPT ensemble) were used. The cutoff radius in the Coulomb interactions was 9.0 Å. An RESPA integrator was set with a time step of 2.0, 2.0, and 6.0 fs, respectively, for bonded interactions and near and far interactions. Trajectories were saved at 500 ps intervals for analysis.

To analyze interactions between ligands and PDE9, the Simulation Interaction Diagram tool and rmsd/RMSF evaluation were used. When ligand–protein contacts were evaluated, a contact strength threshold value of 30% was considered for all of the analyses.

### Chemicals and Biological Materials

Tissue culture reagents were obtained from Gibco-BRL (San Giuliano Milanese, MI, Italy) and Sigma (St. Louis, MO, USA). PF-0447943was purchased from Sigma (St. Louis, MO, USA). The synthesis of DB987 was previously reported by our group.^45^

### Assessment of PDE9 Inhibitory Activity

To assess the inhibitory activity of DB987 against full-length recombinant human PDE9 (PDE9A, SignalChem, Richmond, Canada), the PDE-Glo phosphodiesterase assay (Promega Corp., Madison, WI, USA) was used as previously reported.^29^ The compound was dissolved in DMSO and mixed with the PDE-Glo reaction buffer at a v/v ratio of 1:5. Aliquots of PDE-Glo reaction buffer with appropriate amounts of human recombinant PDE9 were placed in separate wells of a 96-well plate (PerkinElmer, Milan, Italy). Subsequently, the solution (5 μL) containing a PDE inhibitor and 12.5 μL of cGMP solution as a substrate were added to each well. Each time after the addition of the solutions, the plates were gently shaken on a 3D rotator to ensure that the content was evenly distributed over the bottom of each well (PS-M3D Variable Speed/Angle, Multi-Function 3D Rotator, Kisker Biotech GmbH & Co. KG, Steinfurt, Germany). The samples were incubated for 90 min at room temperature. Then, PDE-Glo Termination Buffer and PDE-Glo Detection Solution (12.5 μL) were added to each well. After incubation (20 min, room temperature) and an addition of Kinase-Glo Reagent (50 μL) to each well, the luminescence of each sample was measured by a microplate scintillation and luminescence counter reader TopCountNXT (Packard, Ramsey, MN, USA). IC_50_ values were estimated through nonlinear regression using Prism v.8 for Windows (GraphPad Software, San Diego, CA, USA). Experiments were performed in eight replicates.

### Animals

Male and female Wistar rat pups (7–9 days old) were purchased from Charles River (MI, Italy). Animals were housed at 23 ± 1 °C under a 12 h light–dark cycle (lights on at 07:00) and were fed a standard laboratory diet with ad libitum access to water. The experimental protocols were approved by the Animal Care Committee of the Department of Health Sciences, University of Florence (17E9C.N.GSO/2021).

The experimental procedures were conducted in accordance with the ARRIVE guidelines and were authorized by the Italian Ministry of Health. The ethical policy of the University of Florence complies with Directive 2010/63/EU of the European Parliament and with Italian Regulation DL 26/2014 on the protection of animals used for scientific purposes. According to the law, all efforts were made to fulfill the principle of 3 Rs.

### Preparation of Rat Organotypic Hippocampal Slice Cultures

Organotypic hippocampal slice cultures were prepared as previously reported.^62^ Briefly, hippocampi were removed from the brains of 7–9 day-old Wistar rat pups (Charles River, Milan, Italy), and transverse slices (420 μm) were prepared using a McIlwain tissue chopper and then transferred onto 30 mm-diameter semiporous membranes inserts (Millicell-CM PICM03050; Millipore, Milan, Italy; four slices per insert), which were placed in six-well tissue culture plates containing 1.2 mL of medium per well. The culture medium compound is composed of 50% Eagle’s minimal essential medium, 25% heat-inactivated horse serum, 25% Hanks’ balanced salt solution, 5 mg/mL glucose, 2 mM l-glutamine, and 3.75 mg/mL amphotericin B. Slices were maintained at 37 °C in an incubator in an atmosphere of humidified air and 5% CO_2_ for 2 weeks. During the period of incubation, the slices become mature and ready for the experiments. The slices were incubated for 24 h with 5 μM kainic acid (KA) in the presence or absence of PDE9 inhibitors. KA concentration and incubation time were chosen based on previous studies, and a selective damage was induced in CA3.^30,34,63^ The slices were incubated for 24 with PF-0447943 (0.01–1 μM) or DB987 (0.01–10 μM), alone or in combination with 5 μM KA. Cell death was evaluated using the fluorescent dye propidium iodide (PI, 5 μg/mL), and fluorescence was viewed using an inverted fluorescence microscope (Olympus IX-50; Solent Scientific, Segensworth, UK) equipped with a xenon arc lamp, a low-power objective (4×), and a rhodamine filter. Images were digitized using a video image obtained by a CCD camera (Diagnostic Instruments Inc., Sterling Heights, MI, USA) controlled by software (InCyt Im1TM; Intracellular Imaging Inc., Cincinnati, OH, USA). Images were analyzed by using morphometric analysis software. Cellular death induced by KA in the CA3 hippocampal subfields was quantified by the image software (ImageJ; NIH, Bethesda, MD, USA) detecting the optical density of PI fluorescence (the fluorescence measured in KA-exposed slices in the CA3 region was taken as 100%).^30,33^

### Evaluation of PDE9 Gene Expression by Quantitative Real-Time Polymerase Chain Reaction

Total RNA was isolated from organotypic hippocampal slices (four slices for the sample) using Trizol reagent (Life Technologies, Carlsbad, CA, USA). 1 μg of RNA was retrotranscribed using iScript (Bio-Rad, Milan, Italy). Real-time polymerase chain reaction (RT-PCR) was performed as reported.^64,65^ The following primers were used: PDE9 rat: forward 5′-TCAGAGCGAACTCCCTACAAGGTGAG-3′ and reverse 5′-CTCCACCACTTTGAGTCCTTCCAATTCC-3′; 18S rat: forward 5′-GGCGGCTTTGGTGACTCTAGATAACC-3′ and reverse 5′-CCTGCTGCCTTCCTTGGATGTGG-3′.

### Western Blot Analysis

Western blotting was conducted as previously reported.^34^ Four slices of the sample were dissolved in 1% SDS. BCA (bicinchoninic acid) protein assay was used to quantify the total protein levels. Lysates (20 μg/lane of protein) were resolved by electrophoresis on a 4–20% SDS-polyacrylamide gel (Bio-Rad Laboratories, Hercules, CA, USA) and transferred onto nitrocellulose membranes. After blocking, the blots were incubated overnight at 4 °C with rabbit-polyclonal anti-PDE9 (Millipore, Burlington, Massachusetts, USA) diluted 1:1000 in TBS-T containing 5% nonfat dry milk. β-Tubulin was used as a loading control (monoclonal antibody) purchased from Sigma (St Louis, MO, USA). Immunodetection was performed with HRP-conjugated secondary antibodies (1:2000) antimouse or antirabbit IgG from donkey (Amersham Biosciences, Little Chalfont, UK) in TBS-T containing 5% nonfat dry milk. After the membranes and reactive bands were washed, they were detected using chemiluminescence (ECLplus; Euroclone, Padova, Italy). Quantity One analysis software was used for quantitative analysis (Bio-Rad, Hercules, CA, USA). Results are presented as the mean standard error of the mean (SEM) of different gels and expressed as AU-, which depicts the ratio between the levels of target protein expression and β-tubulin normalized to basal levels.

### Fluorescence Immunohistochemistry

At the end of treatments with KA, the organotypic hippocampal slices were harvested and fixed overnight (O/N) in ice-cold paraformaldehyde (4% in PBS). The following day, slices were placed for at least 2 days in a sucrose solution (18% in PBS) and finally conserved in antifreezing solution at −20 °C until the usage for immunohistochemistry. Immunohistochemistry was performed with the free floating method as previously reported.^30,66,67^

First day: Hippocampal slices were placed in a multiwell and incubated for 60 min with blocking buffer (BB) containing 10% normal goat serum. The slices were then incubated overnight at 4 °C under slight agitation with a mouse anti-NeuN to immunostain neurons (1:400 in BB; product code #MAB377, Millipore, Billerica, MA, USA) and a rabbit anti-PDE9 antibody to immunostain PDE9 (1:100; code ABN32, Merck, Darmstadt, DE), dissolved in BB.

Second day: Slices were incubated for 2 h at room temperature in the dark with AlexaFluor 555 donkey antimouse (1:400 in BB; product code #A31570, Thermo Fisher Scientific, Waltham, MA, USA). Slices were then incubated for 2 h at room temperature in the dark with AlexaFluor 555 donkey antimouse plus AlexaFluor 488 donkey antirabbit (1:400 in BB; product code #A21206, Thermo Fisher Scientific). Astrocytes were immunostained using a mouse anti-GFAP antibody conjugated with the fluorochrome Alexa Fluor 488 for 2 h at room temperature in the dark (1:500; code MAB3402X, Millipore). The slices were mounted onto gelatin-coated slides using Vectashield mounting medium with DAPI (Product Code #H-1200, Vectashield, Burlingame, CA, U.S.A.). Slices were observed under a LEICA TCS SP5 confocal laser scanning microscope (Leica Microsystems CMS GmbH, Mannheim, Germany) equipped with 20×, 40×, and 63× objective (*z* step of 1.2 μm, 0.6, or 0.3 μm, respectively). Confocal scans were acquired keeping all parameters constant.

### Quantitative Analyses for Immunohistochemical Experiments

Image analyses were performed with ImageJ software (NIH, Bethesda, MD, USA) on z-stack projections of the region of interest, corresponding to area CA3. We quantified the number of NeuN-positive neurons in the CA3 Stratum Pyramidalis (SP). PDE9 and GFAP immunofluorescence in CA3 SP were detected setting a fixed threshold level and quantifying the positive pixels above threshold with the ImageJ threshold tool and were expressed as percent-positive pixels/total pixels. Astrocyte branches were measured in accordance with Cerbai et al.^68^ by quantifying only the number of branches longer than 70 μm and expressing their density as branches/mm^2^.

### Statistical Analysis

Data are presented as means ± SEM of *n* experiments. The statistical significance of differences between PI fluorescence intensities data were analyzed using one-way ANOVA with a post hoc Dunnett *w*-test for multiple comparisons. RT-qPCR, western blot, and immunohistochemistry data were statistically analyzed by Student’s *t*-test. All statistical calculations were performed using GRAPH-PAD PRISM v. Eight for Windows (GraphPad Software, San Diego, CA, USA). A probability value (*P*) of <0.05 was considered significant.

## Safety

We did not observe any unexpectedly significant hazards or risks associated with the reported work.

## Supporting Information Available

Additional details on computational studies, such as RMSF and interaction plots, as well as confocal images of CA3 pyramidal neurons and astrocytes (PDF)
