# Supplementary material for: Virtual Screening-Accelerated Discovery of a Phosphodiesterase 9 Inhibitor with Neuroprotective Effects in the Kainate Toxicity In Vitro Model
Source: ACS Chem Neurosci. 2023 Sep 19;14(20):3826–38. doi: 10.1021/acschemneuro.3c00431 (PMC10587872; doi:10.1021/acschemneuro.3c00431)
Supplement: Supplementary file 1 — cn3c00431_si_001.pdf [file cn3c00431_si_001.pdf]

# - SUPPORTING INFORMATION -

Virtual Screening-Accelerated Discovery of a Phosphodiesterase 9 (PDE9) Inhibitor with Neuroprotective Effects in a Kainate Toxicity *in vitro* Model

Elisa Landucci[1°], Giovanni Ribaudo[2°*], Margrate Anyanwu[2], Erika Osselladore[2], Matteo Giannangeli[2], Costanza Mazzantini[1], Daniele Lana[1], Maria Grazia Giovannini[1], Maurizio Memo[2], Domenico E. Pellegrini-Giampietro[1] and Alessandra Gianoncelli[2*]

[1]*Department of Health Sciences, Section of Clinical Pharmacology and Oncology, University of Firenze, Firenze (Italy)*

[2]*Department of Molecular and Translational Medicine, University of Brescia, Brescia (Italy)*

*Corresponding authors: Dr. Giovanni Ribaudo, PhD - Department of Molecular and Translational Medicine, University of Brescia, Viale Europa 11, 25121 Brescia (Italy); tel: +39 030 3717419, email: giovanni.ribaudo@unibs.it; Prof. Alessandra Gianoncelli, PhD - Department of Molecular and Translational Medicine, University of Brescia, Viale Europa 11, 25121 Brescia (Italy); tel: +39 030 3717419, email: alessandra.gianoncelli@unibs.it.

°These authors contributed equally

Computational studies

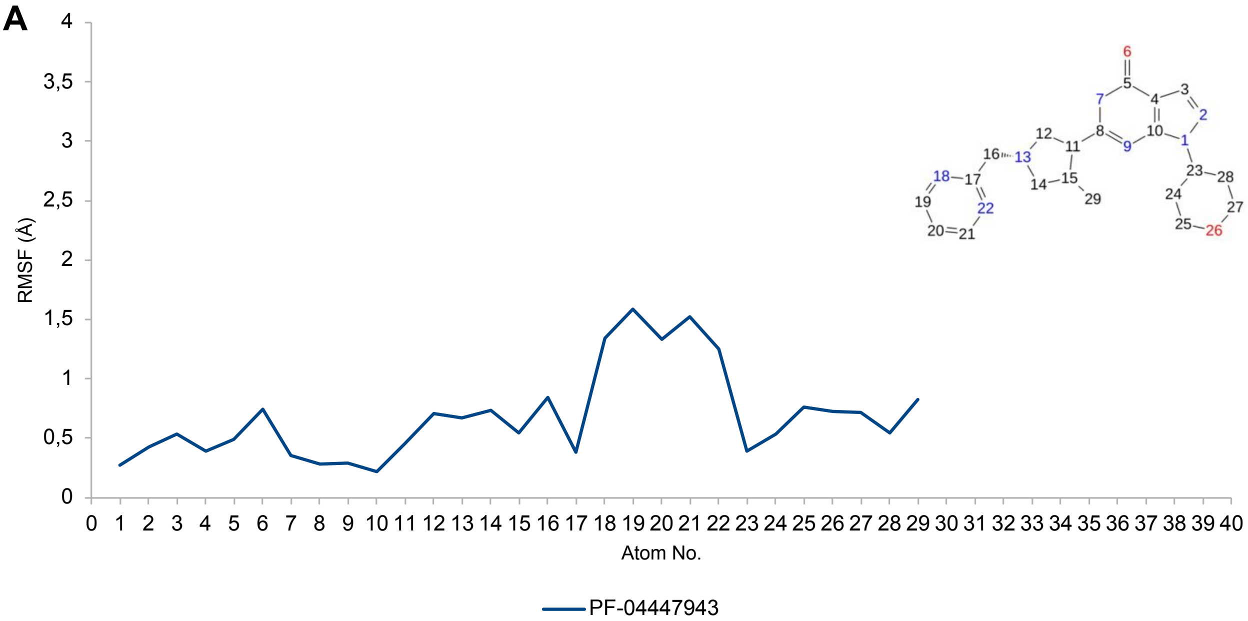

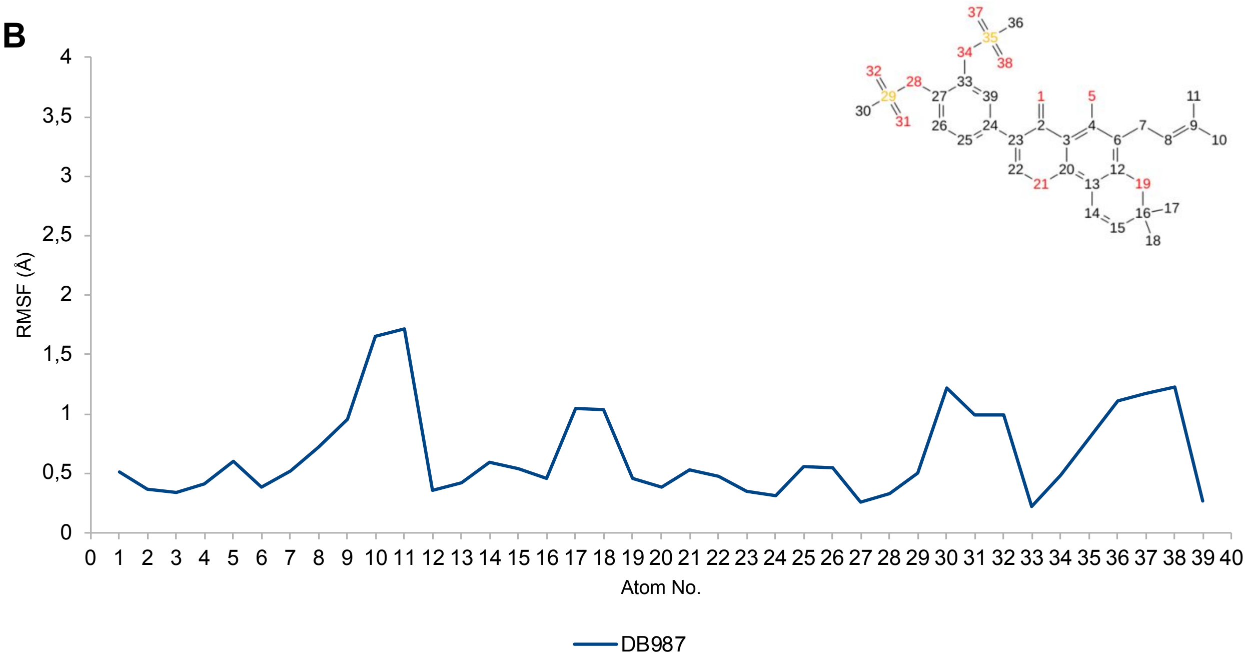

Figure S1. RMSF plots for the studied ligands (A: PF-04447943, B: DB987).

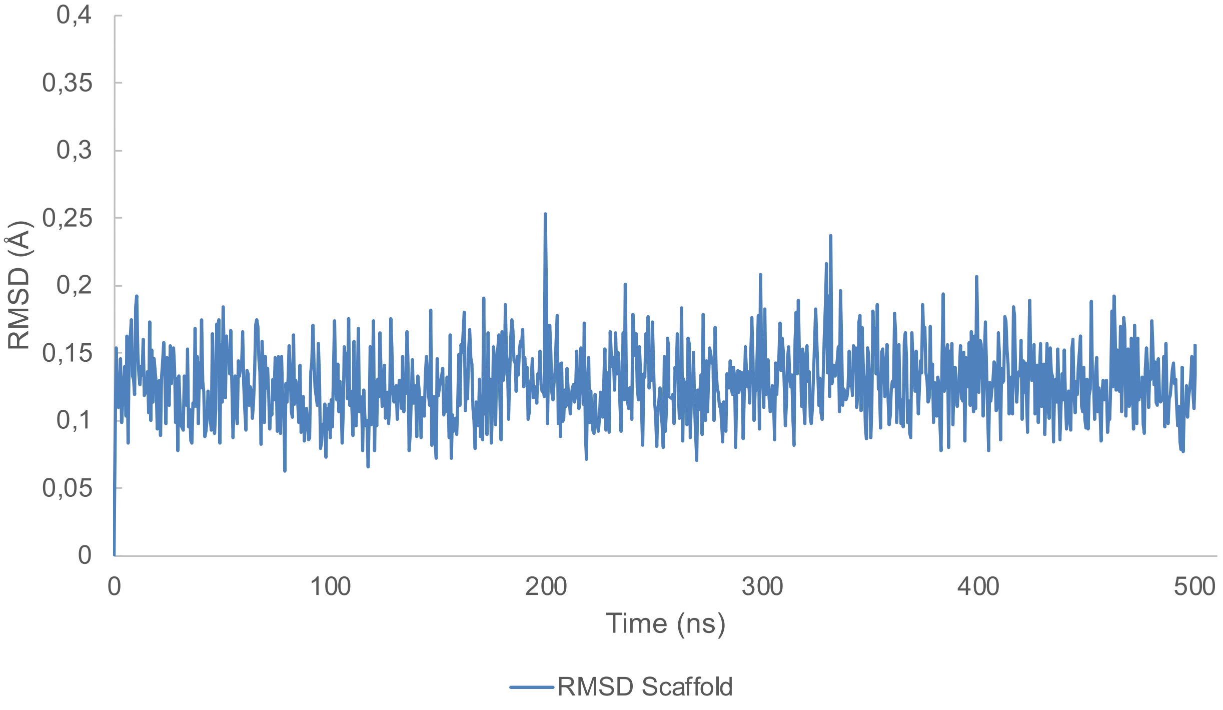

Figure S2. RMSD plot for the scaffold of PF-04447943 (atoms 1-10 - see Figure S1 - aligned with the protein).

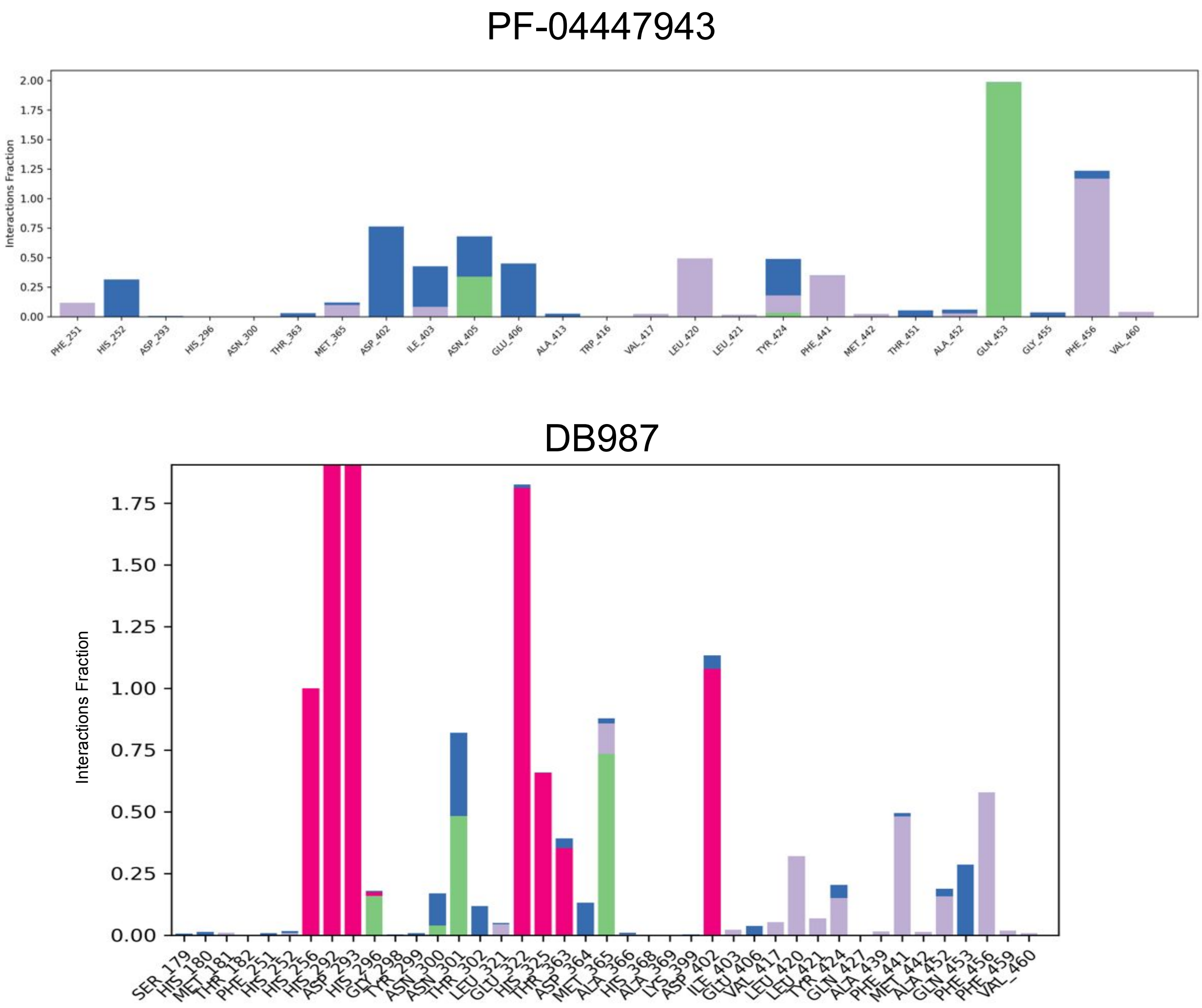

Figure S3. Interaction fraction histograms showing the average number of interactions over the simulation time for the two investigated ligands: hydrophobic interaction (violet), ionic interaction (magenta), water bridged interaction (blue), H-bond (green). Values higher than 1 in the case of multiple contacts between the ligand atoms and the protein.

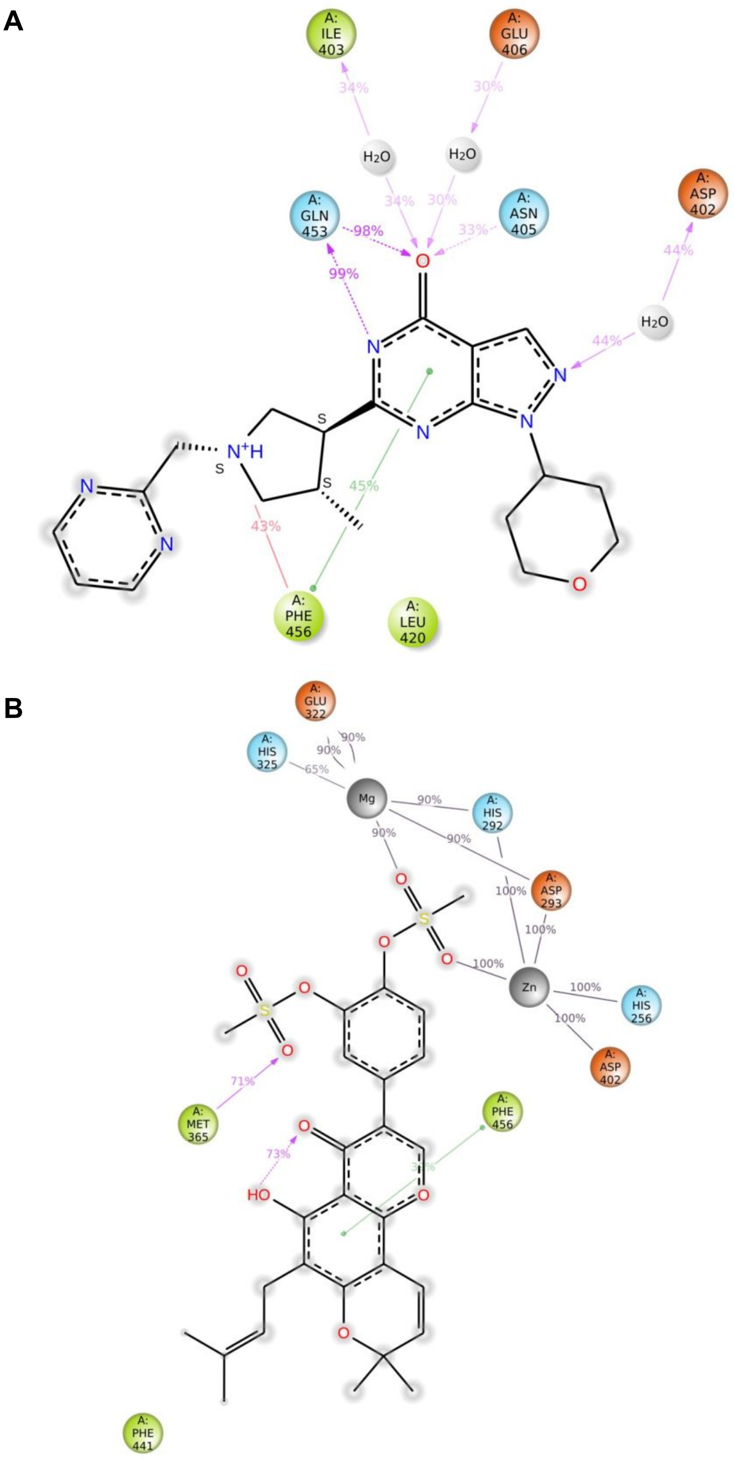

Figure S4. Main interactions established by the studied ligands with PDE9 (A: PF-04447943, B: DB987).

PF-04447943

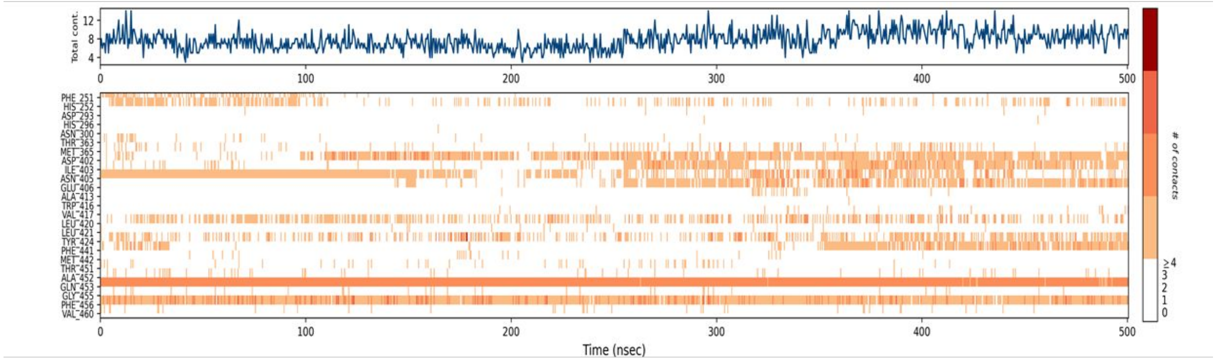

DB987

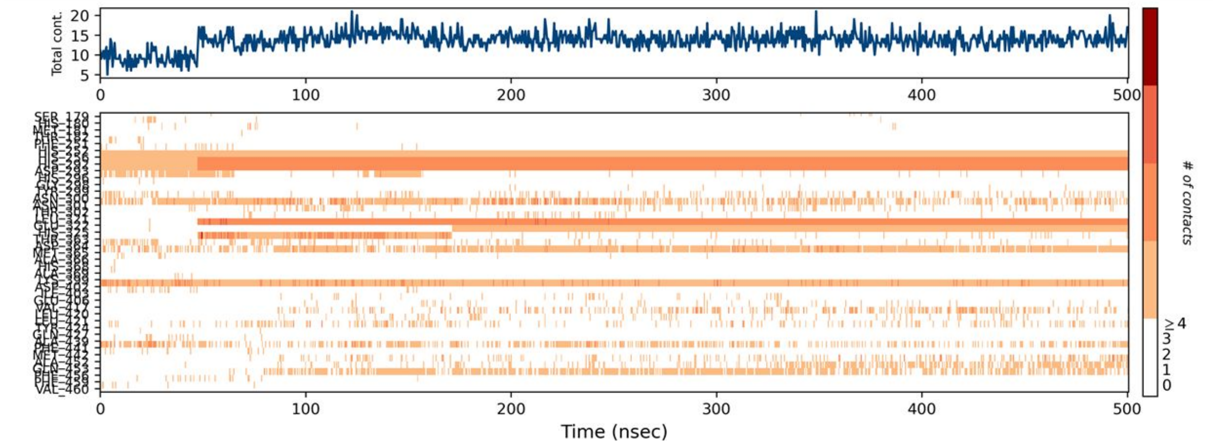

Figure S5. Timeline representation of summarized interactions and contacts (H-bonds, hydrophobic, ionic, water bridges) for the two investigated ligands. The top panel shows the total number of specific contacts the protein makes with the ligand over the course of the trajectory; the bottom panel shows which residues interact with the ligand in each trajectory frame. Some residues make more than one specific contact with the ligand, which is represented by a darker shade of orange, according to the scale to the right of the plot.

Immunohistochemical assessment of astrocytes alteration induced by kainate in CA3 region of organotypic hippocampal slices.

We found that in slices treated with KA astrocytes had significant morphological alterations and decreased GFAP expression. From the qualitative images shown in Supplementary Fig. S4A-B and the magnifications A1 and B1 it is possible to envisage that after KA treatment, astrocytes branches were thinner and shorter than in controls. Indeed, quantitative analysis shown in Supplementary Fig. S4 C demonstrated that the density of branches spanning throughout the SP thickness (longer than 70 μm) was significantly lower (-60%) in KA treated slices than in controls (Student's t test: ***P<0.001 KA vs CTR; CTR, n=10; KA, n=9). Quantitative analyses in Supplementary Fig. 4D-E showed a significant decrease of GFAP expression in both CA3 SP (-45%; Student's t test: ***P<0.001 KA vs CTR; CTR, n=10; KA, n=9) and CA3 SR (-58%; Student's t test: **P<0.01 KA vs CTR; CTR, n=10; KA, n=9) of KA treated slices in comparison to controls.

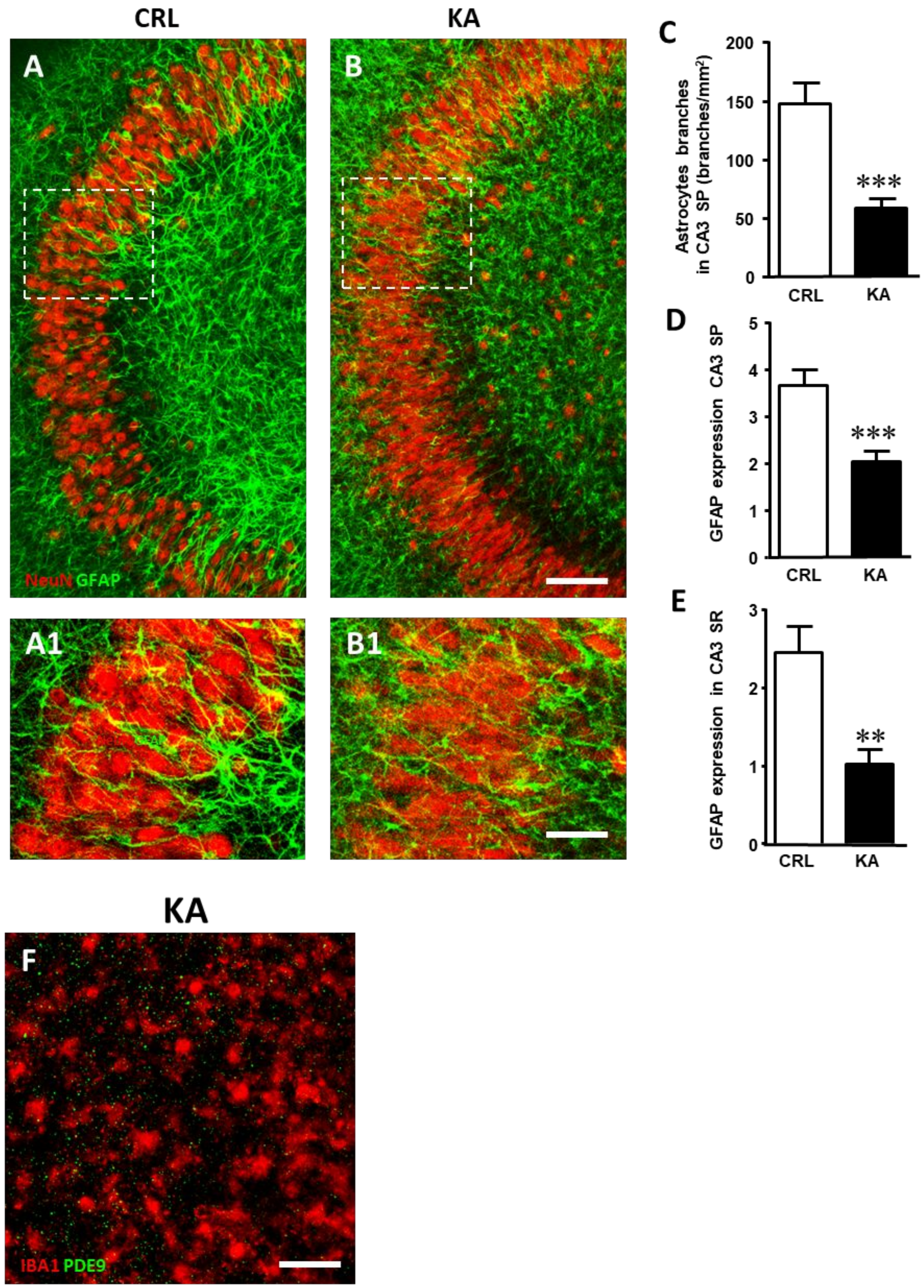

Figure S6. (A-B): Representative confocal images of double fluorescent immunostaining of CA3 pyramidal neurons (NeuN, red) and astrocytes (GFAP, green) in a control (A) and a KA treated (B) slice. Scale bar: 75 µm. (A1,B1): Magnifications of the framed areas of the corresponding slices shown in A and B, respectively. Scale bar: 35 µm. (C): Quantitative analysis astrocytes branches

longer than 70 μm extending through the CA3 SP (CRL n=10, KA n=9). Statistical analysis: Student's t test: ***P<0.001 KA vs CRL. (D): Quantitative analysis of GFAP expression in CA3 SP (CRL n=10, KA n=9). Statistical analysis: Student's t test: ***P<0.001 KA vs CRL. €: Quantitative analysis of GFAP expression in CA3 SR (CRL n=10, KA n=10). Statistical analysis: Student's t test: **P<0.01 KA vs CRL. Bars represent the mean ± SEM of n experiments. (F): Representative confocal image of double fluorescent immunostaining of microglia (IBA1, red) and PDE9 (green) in a KA treated slice. Scale bar: 40 μm.

References

(1) Shang, H.; Hu, Y.; Li, J.; Li, L.; Tian, Y.; Li, X.; Wu, Q.; Zou, Z. The Synthesis and Biological Evaluation of Aloe-Emodin-Coumarin Hybrids as Potential Antitumor Agents. *Molecules* **2022**, *27* (19), 6153. https://doi.org/10.3390/molecules27196153.
(2) Kong, X. B.; Rubin, L.; Chen, L. I.; Ciszewska, G.; Watanabe, K. A.; Tong, W. P.; Sirotnak, F. M.; Chou, T. C. Topoisomerase II-Mediated DNA Cleavage Activity and Irreversibility of Cleavable Complex Formation Induced by DNA Intercalator with Alkylating Capability. *Mol Pharmacol* **1992**, *41* (2), 237–244.
(3) Watanabe, K. A.; Koyama, M. Derivatives of Chryosphanol. US4966918A, October 30, 1990. https://patents.google.com/patent/US4966918A/en?oq=US4966918 (accessed 2023-04-22).
